# Digestive enzymes reduce quality differences between plant and animal proteins: a double-blind crossover study

**DOI:** 10.1186/1550-2783-12-S1-P26

**Published:** 2015-09-21

**Authors:** Julie Minevich, Mark A Olson, Joseph P Mannion, Jaroslav H Boublik, Josh O McPherson, Ryan P Lowery, Kevin Shields, Matthew Sharp, Eduardo O De Souza, Jacob M Wilson, Martin Purpura, Ralf Jäger

**Affiliations:** 1 Department of Health Sciences and Human Performance, The University of Tampa, 401 W. Kennedy Blvd., Tampa, FL 33606, USA; 2 Chemi-Source, Inc., 2665 Vista Pacific Dr., Oceanside, CA 92056, USA; 3 Increnovo LLC, 2138 E Lafayette Pl, Milwaukee, WI 53202, USA

## Background

Whey protein is considered to be the optimal protein source to support muscle protein synthesis (MPS) with resistance training, based on its amino acid content (high in leucine), rapid digestibility, and high bioavailability within the muscle tissue [1]. Athletes can choose from different plant protein sources (e.g. soy, rice, pea, hemp), which differ in numerous ways, such as the presence of allergens (milk, soy), cholesterol, saturated fats, digestion rate (fast, intermediate, or slow absorption of amino acids), or the relative amount of individual amino acids. Rice protein has been shown to promote muscle hypertrophy with resistance training comparable to whey protein [2]. 48g of rice or whey protein isolate immediately post-exercise during an 8-week progressive, non-linear resistance-training protocol increased lean body mass, muscle thickness, and strength with no differences between groups. The findings are likely due to the high dose of protein used in the study, providing amounts of leucine greater than the 1.7 to 3.5g that has been proposed to be the range for optimal MPS. Rice protein, compared to whey (fast) and casein (slow), is an intermediate digesting protein and shows a 6.8% lower total amino acid appearance in the blood [3]. While dairy protein sources contain simple sugars, mainly lactose, plant proteins contain more complex carbohydrates, including fibers and glycoproteins. This study sought to investigate if co-ingestion of a plant protein specific digestive enzyme blend (Digest-All^® ^VP, a proprietary enzyme blend consisting of protease 6.0, protease 4.5, peptidase, bromelain and alpha-galactosidase, Chemi-Source, Inc., Oceanside, CA) can reduce the significant differences in amino acid appearance in the blood between plant and animal proteins.

## Methods

After a 12 hour overnight fast, 11 resistance-trained male subjects (age: 21.4 ± 1.5 years, body weight: 82.5 ± 3.9kg, height: 177.3cm ± 6.1cm, and average training status of 2.3 years ± 1.9 years) were randomly assigned to receive either 60 grams of whey protein concentrate ("WPC", Milk Specialties Global, Eden Prairie, MN), or a 70:30 blend of pea protein (VegOtein^® ^P80, Axiom Foods, Los Angeles, CA) and rice protein (Oryzatein^® ^Silk 80, Axiom Foods, Los Angeles, CA) concentrate ("PRPC"), or PRPC plus Digest-All^® ^VP ("PRPC+DA", Veggie Elite^®^, MRM, Oceanside, CA) in a double-blind, crossover design, separated by a washout period of 7 days. Blood draws were taken immediately prior to, and at 30 minutes, 1, 2, 3, and 4 hours following consumption of WPC, PRPC or PRPC+DA.

## Results

Time to peak (Tmax (min)) for total amino acid (TAA) was faster in the WPC group in comparison to PRPC. However, the addition of digestive enzymes to the plant protein blend increased Tmax of PRPC+DA over WPC (TAA: WPC 62.7 ± 31.3, PRPC 73.6 ± 33.6, PRPC+DA 57.3 ± 24.9). Tmax for the sum of non-essential amino acids (NEAA) showed the same trend: WPC 62.7 ± 31.3, PRPC 73.6 ± 31.1, PRPC+DA 51.8 ± 24.9, while for essential amino acids (EAA) WPC was fastest: WPC 57.3 ± 9.0, PRPC 76.4 ± 28.0, PRPC+DA 70.9 ± 24.3. There were no differences between conditions for Tmax (p = 0.10). Significant differences were detected for AUC (AUC × 10^3 ^[nmol/ml]) whereas the EAA for PRPC 384.5 ± 79.3 was significant lower than WPC 447.1 ± 69.9 (p = 0.002). There were no differences for the AUC between WPC and PRPC+DA 404.9 ± 80.5 (p = 0.16). In addition, no significant differences between conditions were detected for NEAA: WPC 677.5 ± 145.0, PRPC 650.3 ± 192.1, PRPC+DA 643.2 ± 139.8, p = 0.59 and for TAA: WPC 1,187.2 ± 228.3, PRPC 1,071.0 ± 241.0, PRPC+DA 1,083.7 ± 223.0, p = 0.09. There were significant differences between conditions for peak values (Cmax [nmol/ml]) for EAA, whereas WPC (2,261.1 ± 437.2) demonstrated higher values than PRPC (1,797.1 ± 333.4), p = 0.01. There no differences between WPC and PRPC+DA (1,881.4 ± 352.9), p = 0.07. No significance differences in Cmax were found for NEAA (WPC 3,103.4 ± 769.8, PRPC 2,978.2 ± 663.8, PRPC+DA 2,904.8 ± 726.7, p = 0.94) and TAA (WPC 5,694.1 ± 1,317.7, PRPC 4,940.5 ± 951.9, PRPC+DA 4,936.6 ± 1,231.0, p = 0.62).

## Conclusion

Co-ingestion of a plant protein specific digestive enzyme blend (Digest-All^® ^VP) and a pea/rice protein blend increases time to peak, peak concentrations, and amount of amino acid appearance in the blood (AUC) in comparison to pea/rice protein alone, and reduces previously significant differences between WPC and PRPC.
